# Highly Disaggregated Particulate and Gaseous Vehicle Emission Factors and Ambient Concentration Apportionment Using a Plume Regression Technique

**DOI:** 10.1021/acs.est.5c05015

**Published:** 2025-06-04

**Authors:** Naomi J. Farren, Markus Knoll, Alexander Bergmann, Rebecca L. Wagner, Marvin D. Shaw, Samuel Wilson, Yoann Bernard, David C. Carslaw

**Affiliations:** † Wolfson Atmospheric Chemistry Laboratories, 8748 University of York, York YO10 5DD, U.K.; ‡ 27253 Institute of Electrical Measurement and Sensor Systems, Graz University of Technology, Inffeldgasse 33/I, Graz 8010, Austria; § Leverhulme Centre for Climate Change Mitigation School of Biosciences, 7315 University of Sheffield, Sheffield S10 2TN, U.K.; ∥ 482378 The International Council on Clean Transportation, Fasanenstr. 85, 10623 Berlin, Germany

**Keywords:** vehicle emissions, plume sampling, source apportionment, particulates

## Abstract

In this study, vehicle plume measurements from over 27,500 vehicles were made using continuous fast-response instruments located at the curbside for nitrogen oxides (NO_*x*_), particle number (PN), and black carbon (BC) in the city of Milan, Italy. A recently developed *plume regression* technique is further enhanced to calculate highly disaggregated emission factors for a wide range of vehicle classes. The data reveal a strong improvement in the emissions performance for NO_*x*_ from passenger cars on going from laboratory to on-road testing. However, for emissions of PN and BC, disaggregation by vehicle manufacturers for diesel passenger cars highlights anomalously high emissions from some manufacturers. Emissions from one manufacturer, which predate on-road testing, are up to a factor of 4 higher than the average of other manufacturers and are among those being scrutinized in several European countries through enhanced periodic technical inspections (PTI) that for the first time considered PN. Near-road concentration source apportionment reveals a broader range of vehicle types contributing to PN and BC compared to NO_*x*_. The top three contributors to NO_*x*_ concentrations account for 57% of total NO_*x*_ but only 28–29% of total PN and BC. These findings have implications for policies such as low-emission zones of the type adopted in Milan and elsewhere in the world. The combination of curbside measurements and *plume regression* allows for both high-resolution emission measurements and ambient concentration source apportionment.

## Graphical abstract

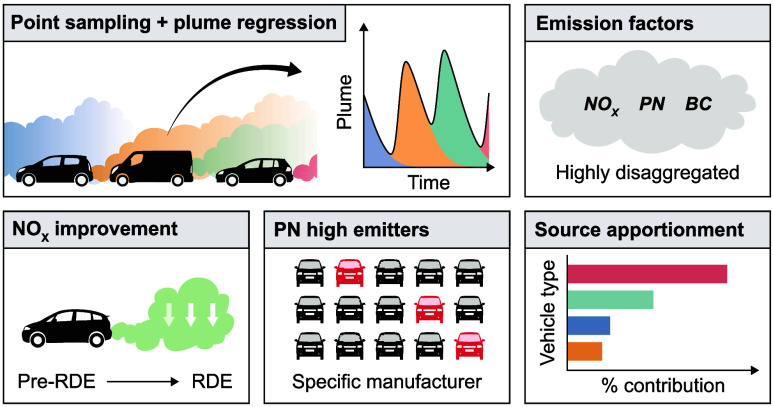

## Introduction

The measurement of real-world vehicle emissions and quantification of contributions to ambient air pollutant concentrations remain a challenging but important topic worldwide. The complexity of this issue is principally related to the nature of the sources themselves, owing to the millions of individual vehicles that move in both space and time. Furthermore, numerous factors influence emissions from vehicles. These include fuel type and vehicle emission control technologies, which have evolved considerably over the past few decades.^1^ Among the other important factors affecting vehicle emissions are how they are driven, maintenance, degradation, tampering, and the wider environment including factors such as road gradient and ambient temperature.^2–4^

Over the past 20 to 30 years, there has been considerable change to how vehicle emissions are regulated and the aftertreatment technologies used to reduce emissions, as described by Wilson et al.^1^ From a regulatory perspective, progressively more stringent emission limits have been set in the EU and UK for gaseous and particulate matter (PM) emissions based on laboratory testing since the early 1990s —so-called ‘Euro standards’.^5,6^ The increasing pressure to reduce vehicle emissions under on-road driving conditions (as opposed to laboratory measurements) led to the introduction of real driving emissions (RDE) testing as part of Euro 6 regulations for passenger cars, during which vehicle emissions are measured by using portable emissions measurement systems (PEMS) under real driving conditions. The introduction of RDE testing greatly expedited the use of advanced aftertreatment technologies for nitrogen oxides (NO_*x*_) control including SCR (Selective Catalytic Reduction), which were required to meet the lowered NO_*x*_ emission limits in a larger range of conditions compared to the laboratory type-approval test.^1^ The vast range of vehicle types, fuels, aftertreatment technologies, and other factors affecting emissions means that it is challenging to quantify emissions behavior for vehicles under actual conditions of use.

While traditional open-path vehicle emission remote sensing is effective for gaseous vehicle emission quantification, the approach is much less well suited for the measurement of particulates.^7,8^ Remote sensing has historically focused on measurements of ‘opacity’ as an indicator of PM emissions.^9,10^ However, the majority of modern diesel and gasoline vehicles are fitted with particulate filters and the opacity-based remote emission sensing measurement is not sensitive enough to accurately quantify PM emissions from current vehicle fleets.^11^ The need to enhance vehicle fuel economy has also led to the widespread use of direct fuel injection techniques in gasoline vehicles, resulting in particle number (PN) emissions being dominated by ultrafine particles.^12^ Furthermore, countries such as The Netherlands, Belgium, Switzerland, and Germany have introduced PN-based tests in their statutory periodic technical inspections (PTI).^13^ PN is also the metric used in the Type Approval process in Europe, where limits are set for both diesel and gasoline vehicles.^14^ It is therefore critical that existing remote emission sensing capabilities are further developed and enable targeted measurement and data processing approaches to more accurately quantify particulate emissions from the transport sector.

In addition to the quantification of vehicular emissions, it is beneficial that the contribution made to ambient concentrations is robustly quantified for pollutants such as nitrogen dioxide (NO_2_) and PM. Of particular interest is the contribution made by traffic-related air pollutants close to roads, where concentrations are highest. However, the quantification of concentrations by source type is highly challenging and is influenced by near-field dispersion processes including vehicle-generated turbulence and complex mixing of plumes in the urban environment.^15,16^ Ideally, information is required about the contributions made by specific vehicle types to ambient concentrations so that effective policies can be developed to reduce emissions and lower pollutant concentrations. This aspect of measurement and analysis capability where information is available on concentration source apportionment is underdeveloped.

In this paper, a recently developed *plume regression* approach is adopted to analyze data from over 27,500 vehicle measurements made in the city of Milan, Italy. The principal aim is to quantify emissions of NO_*x*_, PN and BC based on measurements using instruments that are specialized for gaseous and PM measurements, which extend current remote emission sensing capabilities. Of particular interest is the use of a data analysis approach that reveals both highly disaggregated emissions by vehicle type *and* ambient concentration source apportionment. Furthermore, an important aim is to understand manufacturer-level emissions to identify and quantify anomalously high emissions behavior. Finally, through the quantification of near-road concentration source apportionment, we identify the vehicle categories that contribute most to roadside concentrations of NO_*x*_, PN, and BC.

## Materials and Methods

### Instrumentation

Emission measurements were carried out using the Point Sampling (PS)^17–19^ technique with the system described in Knoll et al.^20^ PS uses fast-response air quality instruments deployed on the curbside to continuously measure vehicle plumes from passing vehicles. An important benefit of PS is that the deployed instrumentation can be selected based on a well-suited measurement technique for each pollutant in question. In this study, high-time resolution (1 Hz) PS measurements were made using three instruments specifically chosen to measure NO_*x*_, carbon dioxide (CO_2_), PN, and BC.

An iterative cavity-enhanced differential optical absorption spectrometer (ICAD), developed by Airyx, was used to measure NO_*x*_ (NO and NO_2_) and CO_2_.^21^ The ICAD relies on optical absorption spectroscopy between 430 and 465 nm to perform direct NO_2_ measurements. Gas phase titration with ozone (O_3_) is used to convert NO to NO_2_, such that total NO_*x*_ can be measured in a second optical cavity; this, in turn, allows for NO concentrations to be derived. The ICAD method is described in more detail in Horbanski et al.^22^ An in-line nondispersive infrared (NDIR) gas sensor provides simultaneous measurements of CO_2_. The instrument response time (*t*_90_) is 2 s at 1 L min^–1^ flow rate.

A custom-designed diffusion charger was used to measure PN concentrations for particles with a diameter greater than 23 nm.^23^ A catalytic stripper can be placed upstream of the diffusion charger to remove the volatile particle fraction as a whole and measure the solid PN concentration only. The catalytic stripper was used in the second part of the campaign from the sixth of October. The measurements reported in this study do not include the use of the catalytic stripper. However, the effect of using the catalytic stripper to remove the volatile fraction on the calculated emission factors is shown in Figure S1. The diffusion charger was calibrated with soot (miniCAST Model 6204 Type B, Jing Ltd.) and NaCl (ATM220, Topas GmbH) in the size range between 23 and 200 nm against a condensation particle counter with a counting efficiency of 1. The diffusion charger sensitivity is approximately 1,000 particles cm^–3^ (1 s, 3σ), and the response time (*t*_90_) is 2.0 s.

A newly developed Black Carbon Tracker (BCT) from the Graz University of Technology was used to measure BC and CO_2_. The device consists of a photoacoustic-based sensor to measure BC and an NDIR sensor to measure the amount of CO_2_. As the sample gas passes through the photoacoustic cell, the periodic thermal excitation of the BC particles by the laser generates a pressure wave that is proportional to the BC mass concentration in the cell. A detailed description is provided in Knoll et al.^24^ The BCT was calibrated with soot (miniCAST Model 6204 Type B, Jing Ltd.) against the factory-calibrated Magee Scientific Aethalometer AE33 (*R* ^2^ > 0.98, sensitivity <2 μg m^–3^). The analyzer has a limit of detection of 1.12 μg m^–3^ (1 s, 3σ) and a response time (*t*_90_) of 0.9 s at a 4.0 L min^–1^ flow rate.

### Measurement Survey

Measurements were conducted in the city center of Milan, Italy, as part of the CARES (City Air Remote Emission Sensing) project.^25^ A total of 27,574 vehicle passes with associated valid vehicle technical data were recorded between 23rd September and 11th October 2021 at Via Madre Cabrini (45.452, 9.199). The PS system was operated unmanned, 24 h per day.^20^ The exhaust plumes of passing vehicles were sampled using two sample lines (Tygon tubing for PN and BC, Teflon tubing for NO_*x*_ and CO_2_), which were positioned at the curbside of the single-lane road and connected to instrumentation housed in a nearby measurement van. A cyclone and impactor were used to measure only particles smaller than 1 μm, and water traps were used to protect the measuring equipment. Light barriers were deployed adjacent to the sampling inlets to record vehicle pass time and vehicle speed and acceleration. An automated number plate recognition (ANPR) system recorded the registration plates of passing vehicles. This information was used to retrieve vehicle technical information including the emission standard, vehicle manufacturer, fuel type, and vehicle registration date. The ambient temperature ranged from 11.9 to 28.2 °C during the measurement period, and the relative humidity was between 30 and 90%. The wind speed ranged from 0.7 to 7.6 m s^–1^ with a median value of 2.5 m s^–1^.

### Plume Measurement and Processing

This study adopts a recently developed approach to the problem of measuring and calculating vehicle emissions that is described in more detail in Farren et al.^19^ The measurements provide 1-Hz time series measurements of NO_*x*_, PN, BC, and CO_2_. Rather than attempt to isolate individual vehicle plumes from the measurements, which is challenging and results in a large fraction of the data not being used, the approach seeks to first quantify the average shape of a CO_2_ plume from all measurements. The plume profile is obtained by averaging the plumes for all vehicle passes where there is at least a 30 s gap between vehicles to avoid plume interference. This approach results in typical plume profiles as shown in Figure 1, which shows the rapid rise in concentration of CO_2_ following a vehicle pass followed by a slower falloff in concentration to a background value. In Figure 1, the concentrations have been normalized such that the area under each plume is equal to one. Normalizing the plume helps with comparing the plume shapes for different pollutants and is convenient for the analysis. Very similar profiles to CO_2_ are observed for NO_*x*_, PN, and BC. While all four species shown in Figure 1 show a similar behavior, the plume profile for CO_2_ was used in the analysis as it is a strong indicator of combustion.

**Figure 1:**
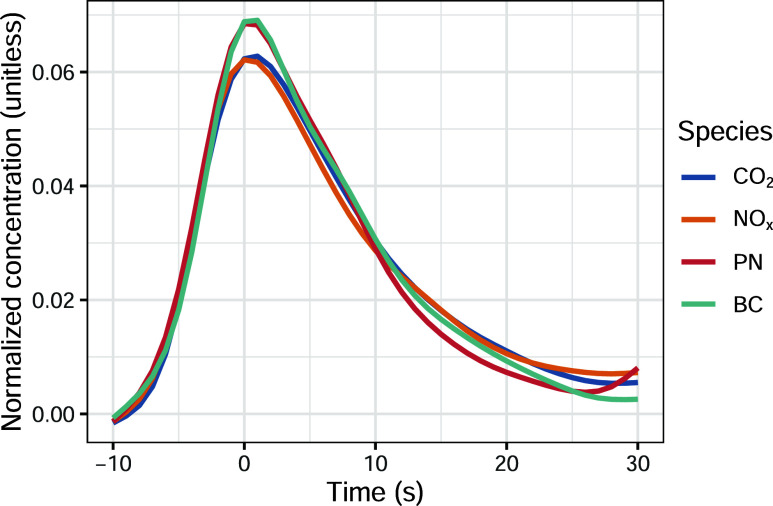
Average plume profiles for CO_2_, NO_*x*_, PN, and BC. The profiles show the rise and fall in pollutant concentration following a vehicle pass at time = 0. The concentrations have been normalized such that the area under each profile is equal to one.

The plume profiles shown in Figure 1 represent the expected rise and fall of a pollutant concentration from a vehicle *on average*. For any individual vehicle pass, plumes are often indistinct and may not be detected at all, for example, due to an unfavorable wind direction. The analysis starts with the definition of the vehicle disaggregation, i.e., the classes of vehicles for which emissions and concentrations need to be quantified. Typically, the classes of vehicles would be fuel and Euro standard categories such as gasoline passenger cars split by Type Approval Category (Euro 3, Euro 4 etc.). However, depending on the question and number of vehicle passes, different aggregations are possible such as splits by the manufacturer. New columns are added to the time series for each vehicle category, with all values initially set to zero. Each time a vehicle from a particular category passes, a normalized plume is added to the column for that vehicle category. The use of a normalized plume means that the sum of a vehicle category column equals the total number of vehicles of that type that have been measured.

The main task of the analysis is to determine the amount by which the plume category weights need to be multiplied by to best explain the concentrations of CO_2_, NO_*x*_, PN, and BC. The concentration considered is the increment above the background, which is determined by applying a rolling second percentile function to the raw data with a window width of 100 s.^26^ The separation of the absolute concentration measurement into a roadside increment and background contribution is useful in its own right and is considered later in the analysis of concentration source apportionment.

In previous work, robust linear regression was used to relate each of the vehicle category weight columns to the increment in concentration above background, which produced results for NO_*x*_ emissions that compared well against independent remote sensing measurements.^19^ In the current work, we use quantile regression to capture the potentially highly skewed emissions distributions for PN and BC.^27,28^ Unlike standard least-squares regression that considers the conditional mean of the response, quantile regression considers the response variable at different quantile levels, τ, where for example τ = 0.5 is the median response. In the context of vehicle emissions, the main benefit is the more robust treatment of data that is highly skewed. We consider both the median response (τ = 0.5) and a high quantile level (τ = 0.95) to explore the skewed nature of the emissions.

The regression coefficients for CO_2_, NO_*x*_, PN, and BC were used to determine molar NO_*x*_/CO_2_, PN/CO_2_, and BC/CO_2_ emission ratios for each class of vehicle considered. For example, the molar NO_*x*_/CO_2_ for the Euro 5 diesel passenger car group is derived by dividing the regression coefficient for that vehicle for NO_*x*_ by the corresponding coefficient for CO_2_. Fuel-specific emission factors (EFs), expressed in grams of pollutant (or number of particles in the case of PN) per kilogram of fuel consumed, were derived from the molar ratios by assuming CO_2_ emission factors of 3.16 and 3.17 kg CO_2_ per kg of fuel for diesel and gasoline vehicles, respectively.^20^ For LPG and CNG, the carbon mass fractions assumed were 3.01 and 2.76. In all cases, it is assumed there is a negligible amount of non-CO_2_ carbon present, e.g., in the form of carbon monoxide, volatile organic hydrocarbons, or PM. A useful benefit of a regression-based approach is that the standard errors are provided for each regression coefficient, from which 95% confidence intervals can be derived. This information enables emission factors to be associated with an uncertainty similar to traditional vehicle emission remote sensing. Because the regression is used to explain the concentration increment, it can be used to provide a direct estimate of the concentration contribution by vehicle type (the concentration source apportionment), as described in detail by Farren et al.^19^

## Results and Discussion

### Road Vehicle Emission Factors

The plume regression approach was applied to the pollutant time series data collected during the Milan measurement campaign and used to generate highly disaggregated EFs for a wide range of vehicle categories. In the case of NO_*x*_ (Figure 2), well-defined EF trends are observed. For example, there is a reduction in NO_*x*_ emissions with an increasing Euro class within each vehicle and fuel type group. As expected, NO_*x*_ emissions from diesel cars of a particular Euro standard are higher than those from their respective gasoline car counterparts. Importantly, the NO_*x*_ EFs compare well with those derived from extensive traditional remote sensing measurements conducted across Europe, which demonstrates the robust nature and accuracy of the plume regression approach.^19^ Indeed, the current work with a sample size of over 27,500 vehicles is more than double that used in the original development of the technique of 11,000 reported by Farren et al., which allows greater disaggregation of emissions. For the first time, it has been possible to investigate emissions from alternatively fueled passenger cars (bifuel LPG and CNG vehicles). In the case of the four Euro classes of LPG vehicles, Figure 2 shows that they emit 1.7 times as much NO_*x*_ as their gasoline equivalents. For CNG passenger cars (only Euro 6 Pre RDE), they emit a factor of 2.2 times more NO_*x*_ than their gasoline equivalents.

**Figure 2:**
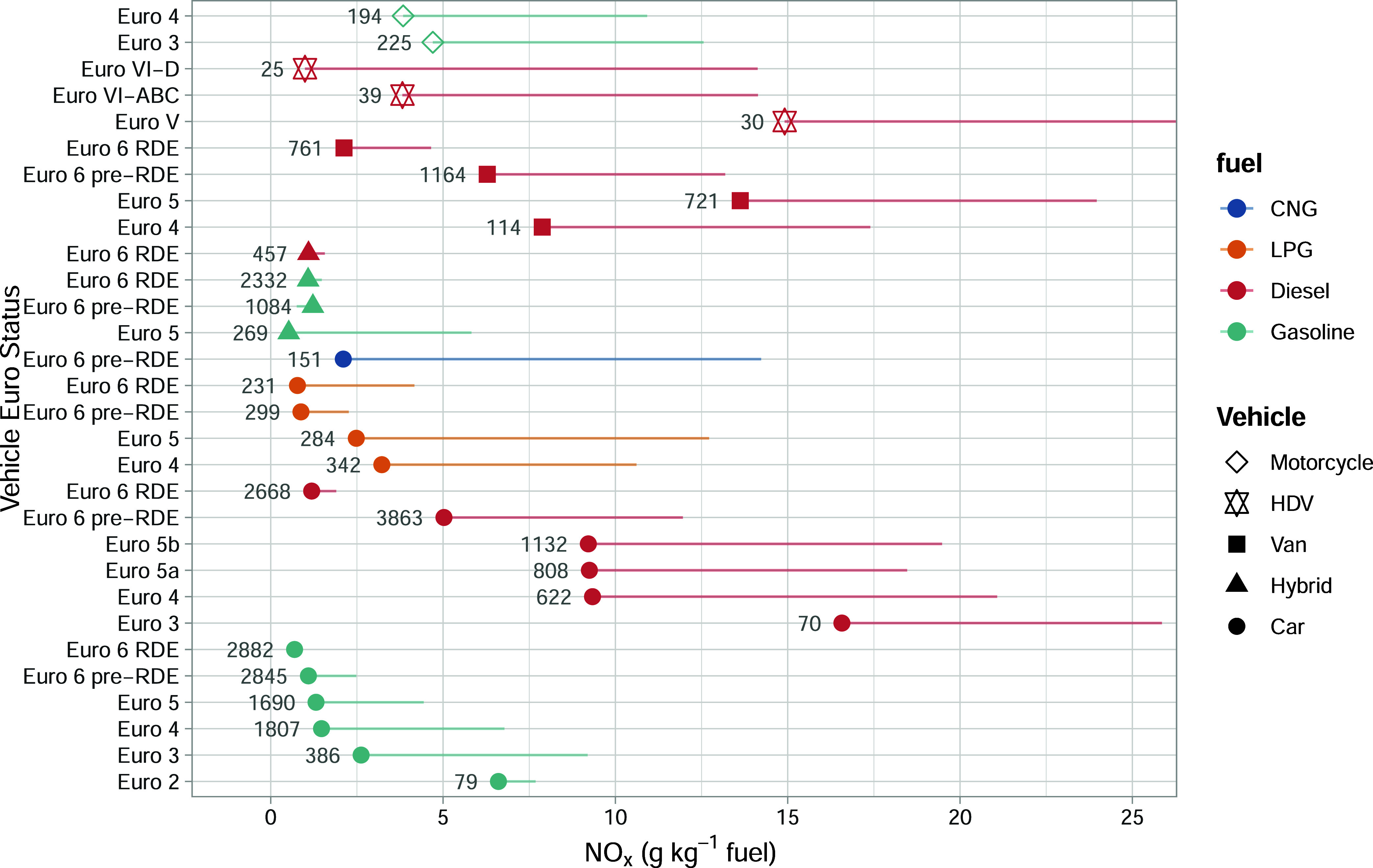
Fuel-specific NO_*x*_ emissions split by vehicle type, fuel, and Euro classification for the Milan data. The numbers next to the data points show the total number of vehicles measured. The symbols show the median (τ = 0.5) response, with the lines extending out to the 0.95 quantile. Note that the ‘RDE’ labeled vehicles are those that have emission limits set based on real driving in addition to laboratory testing.

For particle emissions, the curbside deployment of instrumentation that is specialized for measuring PN and BC mass has led to the generation of PN (Figure 3) and BC (Figure 4) EFs and their associated uncertainties for 30 vehicle categories. This is a significant development over traditional remote emission sensing measurements. In particular, emissions from lower-emission vehicles with diesel particulate filters (DPFs) and gasoline vehicles, which emit even smaller particles, are accurately quantified. Importantly, the EFs from the plume regression approach are generated from *all* of the BC and PN data, which builds upon previous PS studies that require individual plumes to be isolated and therefore suffer from a high proportion of data loss or the requirement for sampling under low traffic conditions.^20,29^ Table S1 provides more information on the emissions of NO_*x*_, PN and BC, fuel-specific EFs, grouped by vehicle type, fuel type, Euro class, and RDE test status.

**Figure 3:**
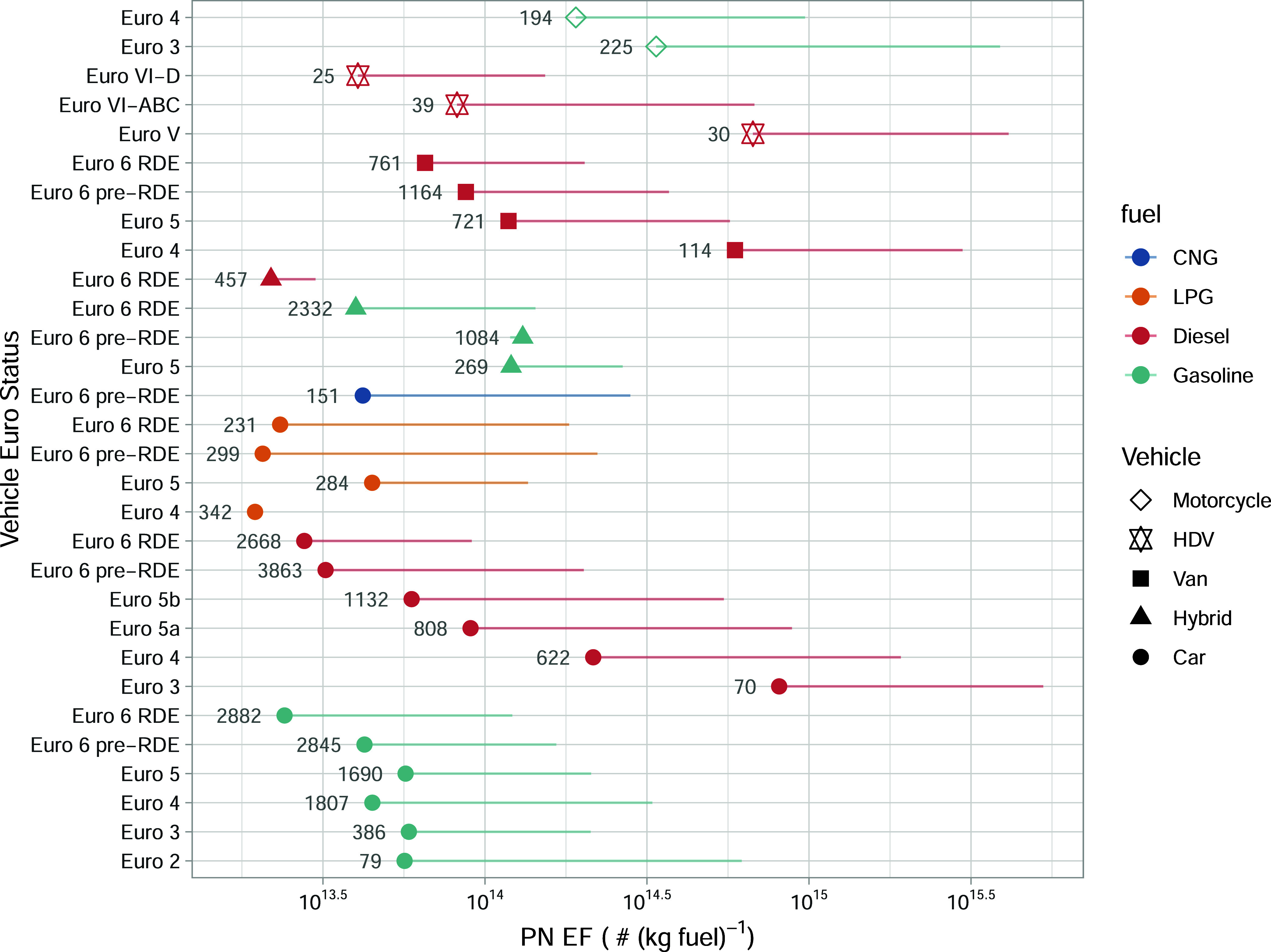
Fuel-specific PN emissions split by vehicle type, fuel, and Euro classification for the Milan data. The numbers next to the data points show the total number of vehicles measured. The symbols show the median (τ = 0.5) response with the lines extending out to the 0.95 quantile. Note that the ‘RDE’ labeled vehicles are those that have emission limits set based on real driving in addition to laboratory testing.

**Figure 4:**
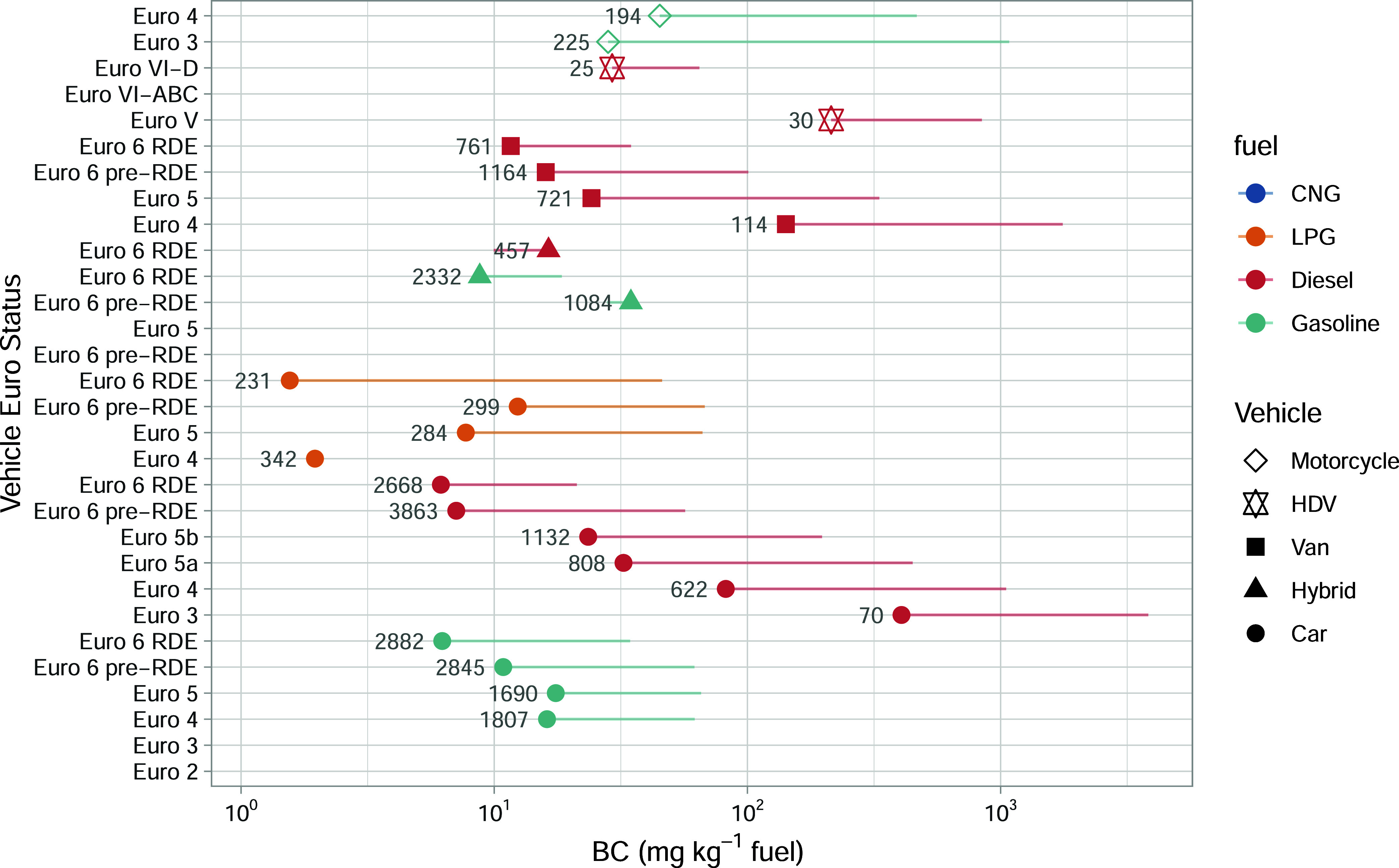
Fuel-specific BC emissions split by vehicle type, fuel, and Euro classification for the Milan data. The numbers next to the data points show the total number of vehicles measured. The symbols show the median (τ = 0.5) response with the lines extending out to the 0.95 quantile. Note that the ‘RDE’ labeled vehicles are those that have emission limits set based on real driving in addition to laboratory testing.

Figure 3 shows that there have been considerable reductions in PN emissions for the different diesel vehicle types across the different Euro standards. Figure 3 splits the diesel car Euro 5 standard between Euro 5a and 5b, where the latter corresponded to the first time a PN limit was set for new models of vehicles in the Type Approval process of 6.0 × 10^11^ km^–1^ in September 2011. The introduction of a PN limit for Euro 5b vehicles effectively made the use of DPF mandatory, although many manufacturers would have introduced DPF vehicles ahead of the introduction of a strict PN limit. However, Figure 3 reveals a clear, progressive improvement in PN emissions for diesel cars from Euro 3 through to the most recent Euro 6 that were tested under Real Driving Emission testing procedures, corresponding to a factor of 25 reduction in emissions. It is also clear that recent technology diesel passenger cars (Euro 6 RDE) are competitive with gasoline cars in terms of PN emissions. There has also been a consistent reduction in PN emissions from vans through the Euro classes, but as Figure 3 shows, the reduction in PN emissions has not been as great as with diesel passenger cars. The improvement in PN emissions in heavy-duty vehicles in going from Euro V (where no PN Type Approval limit existed) to the latter stages of Euro VI (Euro VI-D), where a PN limit of 8.0 × 10^11^ kWh^–1^ was implemented, led to a dramatic reduction in PN emissions by a factor of 36.

PN emissions from gasoline cars have remained fairly stable over the different Euro Standards. There is a significant drop for Euro 6 RDE gasoline cars due to the introduction of the PN limit for direct injection cars (6.0 × 10^11^ km^–1^, same as for diesel) from Euro 6d onward. In a way similar to that for diesel vehicles, this limit value requires the use of particulate filters. Interestingly, the PN emissions of hybrids are higher than those of their combustion-only counterparts, which may be due to engine stop-start and lower engine temperature caused by switching between the electric and combustion engines.^30^ This issue should continue to be monitored as the proportion of hybrid vehicles increases in the fleet. It is also clear that motorcycles are associated with high PN emissions, where current Euro emission legislation lacks a limit value for PN.^31^ Finally, when comparing PN emissions from different vehicle types, it is important to consider the size distribution. Sub-23 nm particles can make up a significant proportion, particularly for CNG and gasoline direct injection vehicles.^32^ This aspect was not considered in this study, as only particles larger than 23 nm were measured. However, this issue needs to be addressed in future studies, especially with the new Euro 7 standard including sub-23 nm particles.

The BC emissions shown in Figure 4 trend to show similar changes by vehicle group as for PN. The clearest changes are seen for diesel vehicles, where the most recent technology Euro 6/VI vehicles show much reduced BC emissions compared with earlier Euro classes. It is interesting to observe that both BC and PN emissions tend to be better controlled in passenger cars compared with vans for the most recent Euro 6 vehicles. PN and BC emissions from Euro 6 vans tend to be a factor of 2 to three higher than passenger cars. This difference might reflect the likely higher mileage of vans compared with passenger cars, but this observation would need to be verified through additional testing and a specific consideration of individual vehicle mileage. As expected, the BC and PN emissions of LPG (and also CNG) cars are lower than those of equivalent gasoline or diesel cars. An interesting measure is the relationship between PN and BC emissions (see Supporting Information in Table S1). On the one hand, it is a quality control measure of how well PN and BC emissions are related. It also provides insight into changes in size distribution or chemical composition due to changing ratios. For diesel and gasoline cars and vans, the ratios generally increase with the newer Euro standards, indicating that either the particles are getting smaller or the BC content is decreasing, which is in good agreement with the literature.^33^ A general aspect is that there are fewer vehicle categories where BC could be determined compared with PN, reflecting the more sensitive measurements of PN.

Figures 2–4 show the range in emissions of NO_*x*_, PN, and BC by considering the median and 0.95 quantile. There is a much greater range of PN and BC emissions than that of NO_*x*_. For example, considering all diesel passenger cars, there is, on average, a factor of 2.0 difference between the 0.5 and 0.95 quantile emissions for NO_*x*_. The range for NO_*x*_ is likely strongly influenced by the well-established differential emissions performance by vehicle manufacturers.^34^ However, for PN, the increase between the two quantile levels is a factor of 7.3 and that for BC a factor of 9.3. These results clearly highlight the highly skewed nature of particle emissions compared with that of NO_*x*_. This finding suggests that there is an important small population of high-emitting vehicles for particulate matter that is not seen for NO_*x*_, and that particulate matter emissions can be increased by several orders of magnitude for high-emitting vehicles. These findings also support the use of quantile regression, which can capture distributions in emissions over other approaches such as ordinary linear regression.

### Manufacturer-Level Emissions

The plume regression approach can further disaggregate emissions beyond fuel type and Euro classification to quantify manufacturer-level emissions when there is sufficient data. Indeed, further splitting of the classes of vehicle to 52 (from 30 used in Figure 3) is a useful test of the plume regression approach and whether it is able to provide useful emissions information at higher levels of disaggregation. Figure 5 shows emissions of NO_*x*_ and PN by manufacturer split by Euro class for Euro 5 (a,b) and newer vehicles, where at least 50 measurements were available. Considering emissions of NO_*x*_, there is an improvement in emissions performance from Euro 5 to 6 (pre-RDE) and Euro 6 (RDE). However, it is clear that for some manufacturers, there was little improvement in NO_*x*_ in going from Euro 5 to Euro 6 (pre-RDE) shown by the overlapping shaded areas in Figure 5. There is a narrow range of low NO_*x*_ emissions for Euro 6 (RDE), demonstrating the robustness of on-road testing as part of the regulation of emissions.

**Figure 5:**
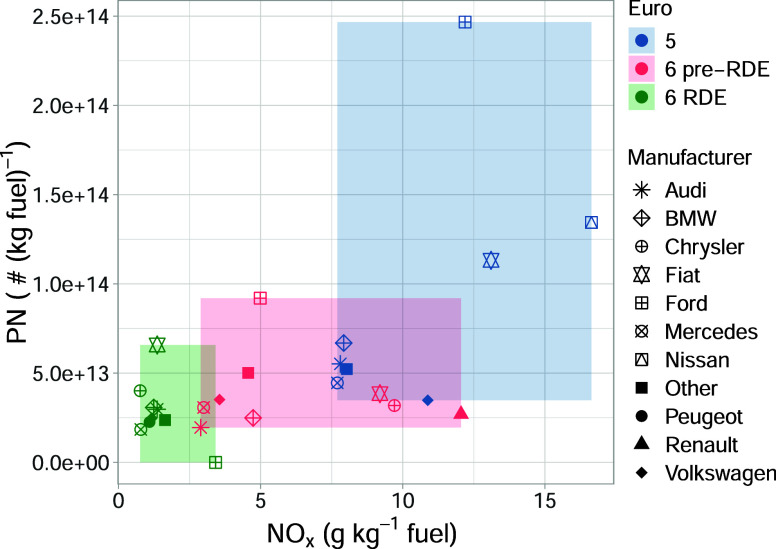
Median emissions of NO_*x*_ and PN for diesel passenger cars by the main vehicle manufacturer groups split by Euro classification. The shaded area shows the range in emissions for the NO_*x*_ and PN for the three Euro classes.

Figure 5 reveals strongly contrasting behavior for emissions of PN. Despite all vehicles considered in Figure 5 having a DPF, the Ford passenger cars are associated with strikingly higher emissions of PN for Euro 5 and 6 (pre-RDE). Euro 5 Ford PN emissions are 4.0 times higher than the median of all other manufacturers of Euro 5 passenger cars and 3.1 times higher than the other manufacturers for Euro 6 pre-RDE. However, for RDE-compliant Euro 6 vehicles, Ford PN emissions are about one-third of that of other manufacturers, suggesting that earlier deficiencies in particle control have been effectively addressed. It is interesting to note that the NO_*x*_ emissions associated with these vehicles are almost three times higher than that of other RDE-compliant vehicles. This observation could be an indication that the engine and aftertreatment strategy was changed to minimize PN emissions at the expense of NO_*x*_ emissions. The anomalously high PN emissions for some Ford diesel cars are also seen for emissions of BC, as shown in Figure S2, which shows the relationship between PN and BC. Similar to the PN results, Euro 5 Ford vehicles have BC emissions 4.2 times higher than the median BC from other manufacturers. Euro 6 RDE Ford diesel passenger cars were also identified as among the higher emitters in a letter to European Type Approval authorities.^35^

Between July 2022 and July 2023, The Netherlands, Belgium, Germany, and Switzerland introduced PN-based tests during the PTIs of diesel vehicles. The PN–PTI is used to identify defective or tampered DPFs. The European Commission recommends that vehicles subject to the PN concentration test should respect the PN–PTI limit of 250,000 particles per cm^3^ when tested during engine idle.^13^ PN–PTI tests in Belgium and preliminary tests in The Netherlands for Euro 5 and 6 diesel vehicles showed that between 6.8 and 15.2% failed the test.^36^ The latest results suggest slightly lower failure rates, but official numbers are yet to be published. The failure rate is highly dependent on the Euro standard, mileage, and age of the vehicle. Interestingly, since the implementation of the PN–PTI, a large number of Ford diesel cars have failed the German “Abgasuntersuchung” (AU) and the Federal Motor Transport Authority (KBA) is investigating Ford for possible defects in DPFs.^37^ Due to pressure from ADAC (the German automobile association) and other organizations, over 7,50,000 Euro 6 diesel cars are affected by a recall worldwide.^38^ Reports have suggested that high PN emissions could be due to poor DPF filter design, leading to hairline cracks in the DPF.

A key benefit common to both PS over traditional remote emission sensing and the new PN–PTI over the PTI opacity test is that PM emissions are measured using a measurement principle with a sufficiently low limit of detection rather than using a plume opacity measurement with limited accuracy at low concentrations. In both cases, this led to the important observation that PN emissions are elevated for a series of diesel passenger cars manufactured by Ford. The combination of emissions monitoring, both during PTI and through roadside measurements, helps to identify anomalies in fleets, identify high emitters, and ensure that emissions are minimized through subsequent repairs.

### Ambient Concentration Source Apportionment

Concentration source apportionment information is of direct relevance to understanding the impacts that vehicles have on ambient air quality, which is not available through traditional remote sensing or plume chase campaigns.^19^ There is increased interest in PN and BC metrics, as PM_2.5_ and PM_10_ are extensively monitored at air quality monitoring stations. But it is evident that more information on PN and BC in the ambient air is required. The *plume regression* method directly estimates the roadside concentration increment above the background that can be associated with all the vehicle categories used as explanatory variables in the regression. Roadside increments in concentration are frequently used in the analysis of air quality data where (for example) the difference between a roadside measurement and a background measurement can be used to define an increment above the background and hence the contribution vehicles on a road make.^39^ The methods used in this study extend this concept and provide detailed information about the different contributions that make up the total concentration increment. Without the development of these methods, it would be necessary to use an air quality model to predict roadside concentrations based on detailed emission factor data. However, predicting concentrations in the near-road environment in urban areas is highly challenging and uncertain owing to the emission factor uncertainty and the complexity of dispersion processes. Methods that provide a direct empirical measure of roadside concentrations are, therefore, highly desirable.

The mean NO_*x*_ concentration observed over the campaign was 70 μg m^–3^ with a derived roadside increment in NO_*x*_ of 33 μg m^–3^ and a background concentration of 36 μg m^–3^ corresponding to a roadside increment of about half the total measured concentration. The roadside increment in NO_*x*_ is therefore a substantial fraction of the total NO_*x*_ concentration and will be typical of many other urban roads, given the importance of road traffic as a source of urban NO_*x*_. In contrast, the PN roadside increment is a much lower fraction of the total concentration of 20% (background = 5392 # per cm^3^ and roadside increment of 1339 # per cm^3^), owing to the many different sources of PN such as brake emissions, secondary aerosol formation, and other urban sources. The roadside increment of 0.9 μg m^–3^ in BC accounted for one-third of the total BC concentration of 2.7 μg m^–3^. These results suggest that there is more scope to reduce roadside concentrations of NO_*x*_ than that of PN or BC through taking action to reduce vehicle emissions.

The concentration contribution made by vehicle exhaust depends on both the absolute exhaust emission strength and the total number of vehicles. Figure 6 shows the concentration source apportionment for NO_*x*_ (top-left panel), PN (top-right panel), and BC (bottom panel). It can be seen from Figure 6 that diesel vehicles (red) dominate total NO_*x*_ concentrations but less so for the concentrations of PN and BC, where gasoline passenger cars (green) make an important contribution. The contribution made by diesel-fueled vehicles to the roadside NO_*x*_ increment is 81%, whereas for PN concentrations, it is 55%, with gasoline being responsible for 42%. Total BC concentrations are dominated by emissions from diesel vehicles, which contribute 61% of the concentration. It is also clear from Figure 6 that for NO_*x*_ concentration contributions, there are fewer vehicles that dominate concentrations compared with PN, where there is a much wider spread in contributions. In the case of PN and BC, it is a broad fleet that contributes rather a small share of vehicles.^40^ For example, the top three contributors to concentrations of NO_*x*_ account for 57% of the total concentration but only 28–29% of total PN and BC concentrations, respectively.

**Figure 6:**
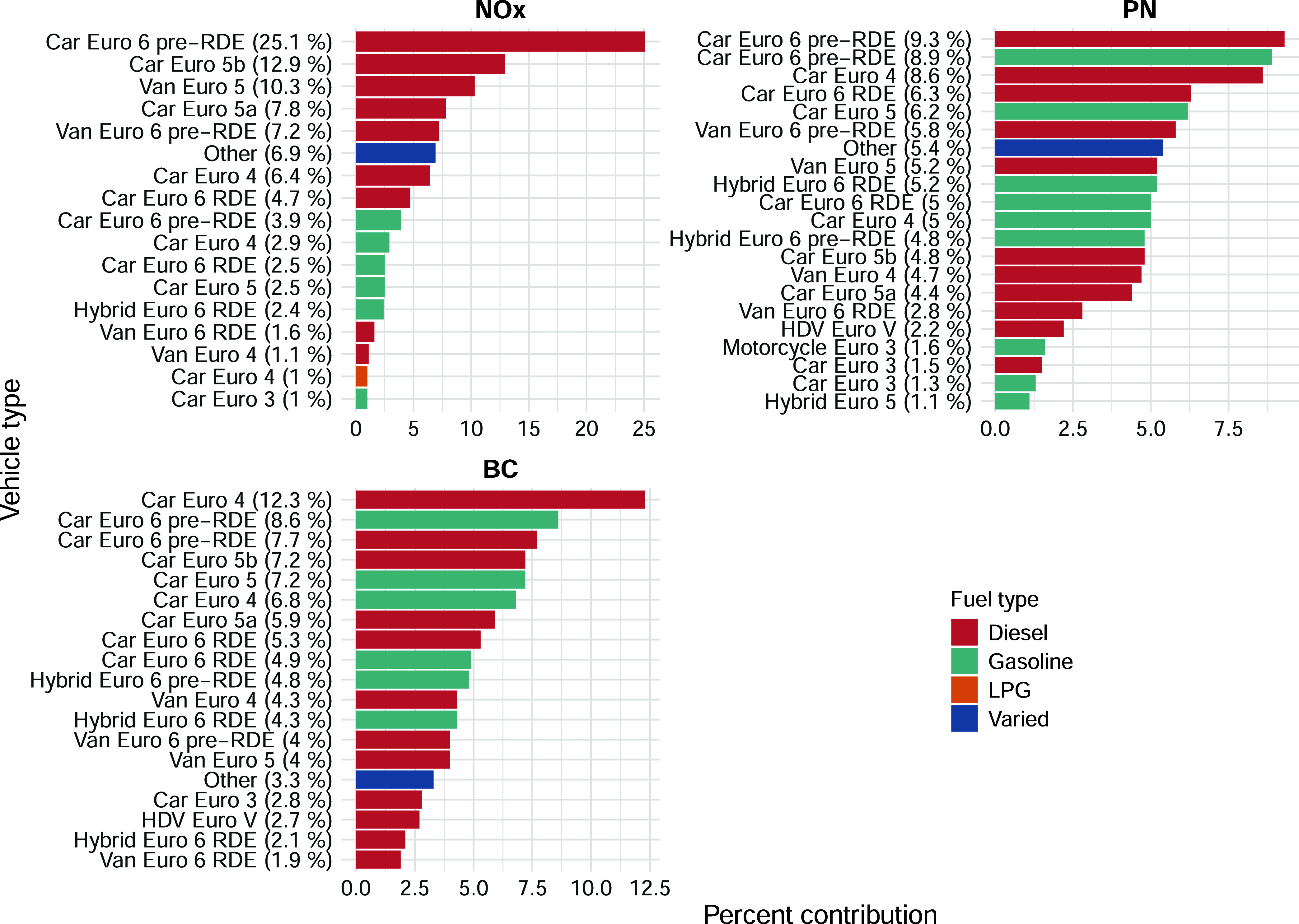
Contributions made to roadside increment concentrations for NO_*x*_, PN and BC in the city center of Milan. The contributions are shown as percentages of the absolute roadside concentration increment. The ‘Varied’ category includes vehicles that contribute less than 1% of the total concentration.

The information in Figure 6 can provide much more detailed information than summaries by fuel type. For example, the contribution of the Euro standard can be directly quantified. Such information is of direct relevance to policies such as low-emission zones of the type that exists in Milan and many other European cities.^41,42^ Ideally, in developing a low-emission zone, it is beneficial to have both fine-grained information on real-world vehicle emissions and *and* information on the corresponding contributions to ambient concentrations. The *plume regression approach* provides this information directly.

In summary, the plume regression approach combined with roadside measurements provides valuable insight into vehicle emission factors and the source apportionment of near-road ambient concentrations. The approach can be applied to a wide range of roadside monitoring locations and pollutants, provided that appropriate sampling and fast-response instrumentation is applied. Such instrumentation (portable and with the required response times and accuracy) is becoming increasingly accessible for a wide range of pollutants; therefore, it is highly feasible to apply this approach to roadside ambient measurement networks. In particular, measuring the particle metrics of PN and BC provides further opportunities to better understand health and climate impacts. It is clear that fast-response roadside measurements are capable of probing the emissions from specific vehicle manufacturers and classes of vehicles, as demonstrated by the identification and quantification of the anomalously high particle emissions from pre-RDE Ford diesel passenger cars. In this respect, fast roadside measurements coupled with the plume regression analysis technique provide a new capability for vehicle market surveillance. By scaling average roadside pollutant increment concentrations by different vehicle fleet compositions, it is possible to generate accurate, data-driven insight into the effectiveness of a vast range of policies designed to improve air quality.

## Supplementary Material



## Notes

Fuel-specific PN emissions with and without the catalytic stripper; emissions of BC and PN for diesel passenger cars by main vehicle manufacturer groups; detailed emissions information for NO_*x*_, PN, and BC with uncertainties in tabular form (PDF)
