# Supplementary material for: Highly Disaggregated Particulate and Gaseous Vehicle Emission Factors and Ambient Concentration Apportionment Using a Plume Regression Technique
Source: Environ Sci Technol. 2025 Jun 4;59(23):11698–707. doi: 10.1021/acs.est.5c05015 (PMC12177929; doi:10.1021/acs.est.5c05015)
Supplement: Supplementary file 1 [file es5c05015_si_001.pdf]

Highly disaggregated particle number and nitrogen oxides vehicle emissions and ambient concentration apportionment

Naomi J. Farren,[†] Markus Knoll,[‡] Alexander Bergmann,[‡] Rebecca L. Wagner,[¶] Marvin D. Shaw,[†] Sam Wilson,[†] Yoann Bernard,[§] and David C. Carslaw[*,†]

†*Wolfson Atmospheric Chemistry Laboratories, University of York, York, YO10 5DD, United Kingdom*

‡*Institute of Electrical Measurement and Sensor Systems, Graz University of Technology, Inffeldgasse 33/I, Graz 8010, Austria*

¶*Leverhulme Centre for Climate Change Mitigation School of Biosciences, University of Sheffield, Sheffield, S10 2TN, United Kingdom*

§*The International Council on Clean Transportation, Fasanenstr. 85, 10623 Berlin, Germany*

E-mail: david.carslaw@york.ac.uk

Summary

Number of pages: 5

Number of Figures: 2

Number of Tables: 1

Supporting Information Available

The following files are available free of charge.

Figures

Figure S1: Fuel-specific PN emissions split by vehicle type, fuel and Euro classification for the Milan data. The lower range shown corresponds to the PN measurement including a catalytic stripper to remove volatile particles (solid PN) and the upper range without the catalytic stripper (total PN). The PN measurement setup was operated without the stripper from 23rd September to 6th October without catalytic stripper and with the stripper after the 6th October.

Figure S2: Emissions of BC and PN for diesel passenger cars by main vehicle manufacturer groups split by Euro classification. The shaded area shows range in emissions for BC and PN for the three Euro classes.

Tables

Table S1: Median NO_x, PN and BC fuel-specific emission factors grouped by vehicle type, fuel type and Euro class with the 95% confidence intervals shown. [NO_x], [PN] and [BC] are the per vehicle mean roadside NO_x, PN and BC increment concentrations respectively. ‘n’ denotes the sample size..

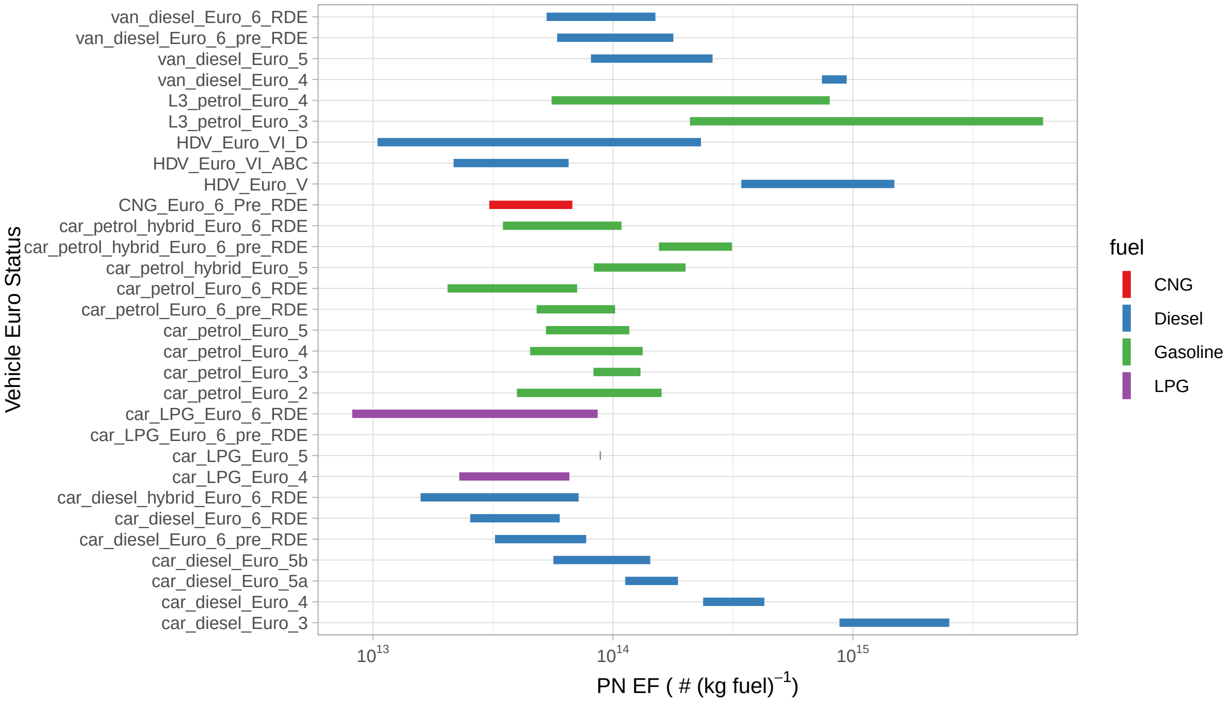

Figure S1: Fuel-specific PN emissions split by vehicle type, fuel and Euro classification for the Milan data. The lower range shown corresponds to the PN instrument using a catalytic stripper to remove volatile particles (solid PN) and the upper range without the catalytic stripper (total PN). From the 23rd September to 6th October the Diffusion Charger was operated without the stripper and with the stripper after the 6th October.

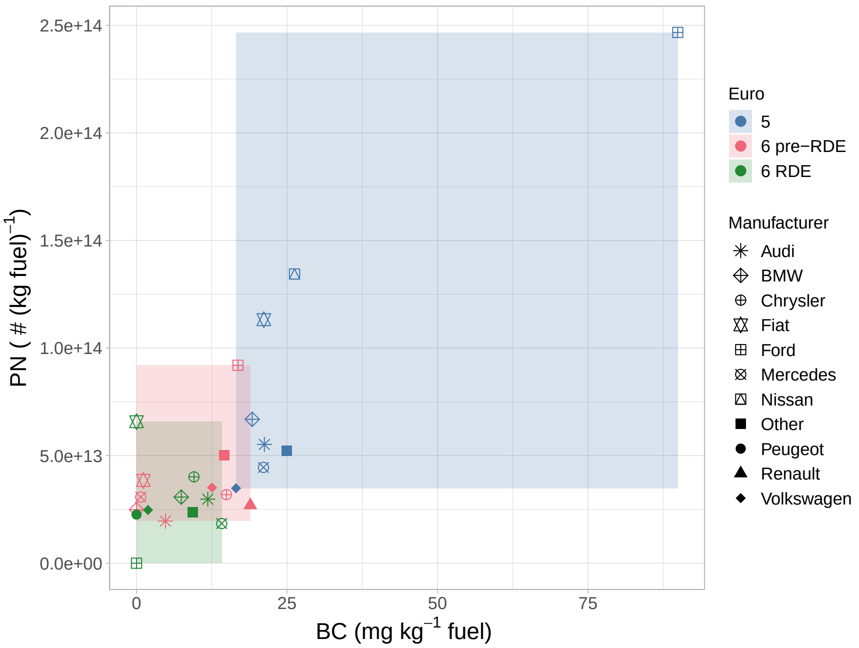

Figure S2: Emissions of BC and PN for diesel passenger cars by main vehicle manufacturer groups split by Euro classification. The shaded area shows range in emissions for BC and PN for the three Euro classes.

Table S1: Median NO_x, total PN and BC fuel-specific emission factors grouped by vehicle type, fuel type and Euro class with the 95% confidence intervals shown. [NO_x], [PN] and [BC] are the per vehicle mean roadside NO_x, PN and BC increment concentrations respectively. PN BC^{-1} is the relation from PN to BC emissions. 'n' denotes the sample size.

**Vehicle**	**Fuel**	**Euro class**	**NO_x EF (g kg^{-1} fuel)**	**PN EF (# 10^{13} kg^{-1} fuel)**	**BC EF (mg kg^{-1} fuel)**	**[NO_x] (ppb)**	**[PN] (# cm^{-3})**	**[BC] (ng m^{-3})**	**PN BC^{-1} (10^{12} mg^{-1})**	**n**
Car	Gasoline	2	6.6 ± 1.6	5.6 ± 1.9	0 ± 10.4	7.75	92	0	-	79
Car	Gasoline	3	2.6 ± 0.6	5.8 ± 1.3	0 ± 4.4	2.04	63	0	-	386
Car	Gasoline	4	1.5 ± 0.1	4.5 ± 0.5	16.2 ± 2.8	1.27	54	19	2.78	1807
Car	Gasoline	5	1.3 ± 0.1	5.7 ± 0.6	17.5 ± 3.1	1.18	71	22	3.26	1690
Car	Gasoline	6 pre-RDE	1.1 ± 0.1	4.2 ± 0.3	10.9 ± 1.8	1.11	60	15	3.85	2845
Car	Gasoline	6 RDE	0.7 ± 0.1	2.4 ± 0.2	6.2 ± 1.7	0.69	33	9	3.87	2882
Car	Diesel	3	16.6 ± 8.8	80.9 ± 35.8	405.9 ± 199	6.03	409	205	1.99	70
Car	Diesel	4	9.3 ± 1.3	21.6 ± 3.1	82.1 ± 11.6	8.24	265	101	2.63	622
Car	Diesel	5a	9.2 ± 0.9	9 ± 1.2	32.4 ± 5.8	7.69	104	37	2.78	808
Car	Diesel	5b	9.2 ± 0.6	5.9 ± 0.7	23.5 ± 3.8	9.13	82	32	2.51	1132
Car	Diesel	6 pre-RDE	5 ± 0.2	3.2 ± 0.2	7.1 ± 1.5	5.19	46	10	4.51	3863
Car	Diesel	6 RDE	1.2 ± 0.1	2.8 ± 0.2	6.2 ± 1.4	1.40	45	10	4.52	2668
Car	LPG	4	3.2 ± 0.6	2 ± 0.8	2 ± 5.2	2.45	21	2	10.00	342
Car	LPG	5	2.5 ± 0.5	4.5 ± 1.1	7.7 ± 6.5	2.48	62	11	5.84	284
Car	LPG	6 pre-RDE	0.9 ± 0.3	2.1 ± 1	12.4 ± 7.3	0.72	23	14	1.69	299
Car	LPG	6 RDE	0.8 ± 0.2	2.3 ± 0.8	1.6 ± 5.6	0.83	35	2	14.38	231
Car	CNG	6 pre-RDE	2.1 ± 0.4	4.2 ± 0.8	0 ± 4.4	2.84	78	0	-	151
Hybrid	Gasoline	5	0.5 ± 0.3	12 ± 3.6	0 ± 10.4	0.24	76	0	-	269
Hybrid	Gasoline	6 pre-RDE	1.2 ± 0.2	13.1 ± 1.7	34.7 ± 8	0.57	85	22	3.78	1084
Hybrid	Gasoline	6 RDE	1.1 ± 0.1	4 ± 0.4	8.8 ± 2.5	0.83	42	9	4.55	2332
Hybrid	Diesel	6 RDE	1.1 ± 0.2	2.2 ± 0.6	16.4 ± 4.5	1.13	31	23	1.34	457
Van	Diesel	4	7.9 ± 2.4	59.1 ± 13.6	141.9 ± 44.9	7.66	798	192	4.16	114
Van	Diesel	5	13.6 ± 1.3	11.8 ± 1.5	24.2 ± 5.6	11.40	137	28	4.88	721
Van	Diesel	6 pre-RDE	6.3 ± 0.6	8.7 ± 1	16 ± 4.1	4.95	96	17	5.44	1164
Van	Diesel	6 RDE	2.1 ± 0.3	6.5 ± 0.9	11.6 ± 4.9	1.65	70	13	5.60	761
HDV	Diesel	V	14.9 ± 5.5	67.1 ± 33.2	214.2 ± 106.3	22.73	1420	453	3.13	30
HDV	Diesel	VI-ABC	3.8 ± 1.3	8.2 ± 2.7	0 ± 3.4	10.18	303	0	-	39
HDV	Diesel	VI-D	1 ± 0.4	4.1 ± 4.4	29.2 ± 12.1	1.99	113	81	1.4	25
Motorcycle	Gasoline	3	4.7 ± 2.3	33.8 ± 12.7	28.2 ± 27.7	1.34	133	11	11.99	225
Motorcycle	Gasoline	4	3.8 ± 1.4	19.1 ± 6.3	45.1 ± 26.4	1.34	93	22	4.24	194
